# Patterns of News Consumption during the COVID-19 Pandemic Crisis: A 2.5 Year Longitudinal Study in the Netherlands

**DOI:** 10.1080/1461670X.2024.2407944

**Published:** 2024-09-27

**Authors:** Adriana Solovei, Julia C.M. van Weert, Bas van den Putte, Mark Boukes, Toni G.L.A. van der Meer, Saar Mollen, Eline S. Smit, Nida Gizem Yilmaz, Marijn de Bruin

**Affiliations:** a Department of Communication Science, Amsterdam School of Communifcation Research/ASCoR, University of Amsterdam, Amsterdam, The Netherlands; b Corona Behavioural Unit, National Institute for Public Health and the Environment (RIVM), Amsterdam, The Netherlands; c IQ Healthcare, Radboud Institute of Health Sciences, Radboud University Medical Center, Amsterdam, The Netherlands

**Keywords:** News consumption, media channels, COVID-19, crisis communication, pandemic preparedness, health communication

## Abstract

During major long-term crises, such as the COVID-19 pandemic, news media are crucial sources of information for the public. This study aimed to explore the frequency of COVID-19-related news consumption based on (1) phase of the pandemic, (2) socio-demographic characteristics, and (3) news information channels. The study used a dynamic cohort design with 18 rounds of data collection, including 306,692 responses from 83,180 unique respondents in the Netherlands from 17 April 2020 to 11 September 2022. Results showed that the frequency of general COVID-19-related news media consumption varied throughout the pandemic, following a general decreasing trend with relative spikes often coinciding with periods of stricter behavioural regulations. TV news, newspapers, and online news websites were the most popular news information channels among the respondents. Furthermore, multilevel regression analyses identified several socio-demographic factors influencing news consumption and preferred channels, namely age, migration background, living status, health status, and trust in government — these results remained stable throughout the pandemic. This study highlights the importance of selecting appropriate news channels to effectively reach different socio-demographic groups and shows that during a prolonged crisis, news consumption about the crisis fluctuates with worsening conditions but generally follows a decreasing trend.

## Introduction

In disturbing and unstable times, such as public health crises, citizens generally have a high need for information regarding the latest (epidemiological) developments and corresponding preventive behavioural regulations (Anwar et al. 2020; Sachs et al. 2022). Moreover, complex crises demand a timely reach of the public with the right information, to prevent further escalations of the problems (Parmer et al. 2016). News media are key sources that can fulfil this need for information in times of crisis (McCombs and Weaver 1973; van der Meer 2018). Empirical evidence in the context of crisis communication has suggested that news consumption in such turbulent times can differ between population segments, for example depending on their demographic characteristics (van Aelst et al. 2021) or trust in the entity managing the crisis (Glik 2007), indicating that some (potentially important) crisis-related information may not timely reach certain population groups. This highlights the importance of understanding news media consumption across different demographic segments during a long-term, multi-faceted crisis. In the current paper, we studied this in the context of the COVID-19 pandemic, one of the most disruptive global health crises of our time, causing worldwide over 770 million infections and 7 million deaths (WHO 2023), along with widespread negative economic and societal consequences (Brodeur et al. 2021). This will provide important insights into how to address the need for information of citizens during future crises.

While several studies have been conducted on the topic of news consumption in the context of the COVID-19 pandemic (for examples see Te Poel et al. 2021; van Aelst et al. 2021; Vermeer et al. 2022), to date, few studies have employed a longitudinal design applied over a timespan of more than two years of the pandemic. A recent study by Knudsen, Nordø, and Iversen (2023) did employ longitudinal analyses up until January 2022, however, focused on general trust in the news media, rather than on mapping out news consumption in more detail, which would allow assessing patterns across different phases of a crisis. This issue is crucial to address because, as previously mentioned, news consumption can vary among different socio-demographic segments throughout a crisis. Consequently, findings from the beginning of a long-term pandemic may not be applicable to the entire crisis period. This gap in the literature, regarding the possible changing patterns in news consumption during different phases of a long-term crisis, is an essential one, because such information is crucial for allowing policymakers, public health professionals, and crisis managers to best reach their target groups during a crisis to keep them informed and stimulate them to adhere to behavioural regulations. In this paper, we aim to address this gap in the literature, by presenting an analysis of 18 survey waves, covering a 2.5-year period from April 2020 to September 2022 (by when most of the pandemic regulations had been given up in the Netherlands), focusing on a large cohort of Dutch citizens.

## Theoretical Background

One of the classic paradigms used to explain news media consumption, including during crises, is uses and gratifications theory (Katz et al. 1974; Ruggiero 2000). This theory states that people actively choose to use specific (news) media sources to satisfy specific needs. Examples of such needs include, amongst others, (a) the need for orientation (i.e., seeking information in order to clarify unknown circumstances) (McCombs and Weaver 1973; van der Meer 2018); (b) need for safety (i.e., seeking information in order to learn how to stay safe or protect others in uncertain situations) (Hu and Zhang 2014; Whiting and Williams 2013); (c) need for diversion and escapism (i.e., seeking information and engaging with media content in order to seek relief from stress and anxiety) (Korgaonkar and Wolin 1999; Palmgreen and Rayburn 1979), (d) the need for maintaining social relationships (i.e., seeking information in order to share it with others, thereby facilitating interpersonal connections; Papacharissi and Rubin 2000), and for many (e) news use is a comforting ritual that functions as part of their daily routines (Groot Kormelink and Meijer 2019).

In the context of COVID-19, Mihelj, Kondor, and Štětka (2022) showed in a multi-country European study based on qualitative interviews and media diaries conducted in the first months of the pandemic that, apart from the abovementioned needs, an important reason why people consumed more news was the fact that, due to the nature of the crisis, they had to spend more time at home, moved in with other family-members who influenced their news media usage and had fewer alternative options for passing time, which altered their regular news consumption habits. This latter motivation supports Rubin’s habitual perspective of news consumption (1984), which suggests that users do not always consume media to satisfy specific information needs, but also because of habits such as pass time, which can be affected by changing circumstances, such as a long-term crisis.

Further building on these insights, Moe, Nærland, and Ytre-Arne (2023) proposed a sequential typology of news consumption during crises, consisting of three main phases. First, in the “ritual check-in” phase, before the start of a crisis, people routinely check the news to confirm stability in their lives. Second, a state of “shocked immersion” occurs, after the onset of the crisis, with individuals seeking comprehensive updates to understand the situation and intensifying their news consumption, for example, by watching livestreams or consulting multiple trusted sources in an effort to regain security. In the third phase, “regained stability”, some time after the onset of the crisis, the public has gained a deeper understanding of the crisis and its implications for themselves, and hence follow the news less intensively than in previous phase, switching instead to, for example, interpersonal communication with others, in order to restore a sense of security and normalcy. Findings from a qualitative study by Groot Kormelink and Klein Gunnewiek (2022) on news consumption patterns during the first COVID-19 wave in the Netherlands generally align with this typology. After an initial surge in news consumption, a phase of reduction or avoidance of consumption of traditional news was noticed, which can be seen as an addition to the sequential typology. However, over time, individuals normalized their news consumption patterns, which is in line with Moe, Nærland, and Ytre-Arne’s (2023) concept of “regained stability”.

Furthermore, quantitative research has also shown that general news consumption increased at the beginning of the COVID-19 pandemic in several countries, compared to the level just before the start of the pandemic (Nielsen et al. 2020; Vermeer et al. 2022). However, as mentioned earlier, there is a lack of (quantitative) studies exploring patterns of news consumption over a longer period of time during the COVID-19 pandemic. Considering the specific character of a pandemic crisis, with waves of infections leading to variations in hospitalizations and deaths over time, it can also be expected that within these main phases news consumption may fluctuate, depending on the epidemiological circumstances of a pandemic. More specifically, in the Netherlands, the COVID-19 pandemic knew four main waves of infection (i.e., March–May 2020; September–December 2020; March–April 2021; October-December 2021), characterized by increased numbers of infections and hospitalizations (RIVM 2023). Moreover, the pandemic stringency index (i.e., composite measure recording the number and strictness of government policies) also varied throughout the crisis (Hale et al. 2021). The start of new waves of infections and more stringent policy regulations have arguably led to an increased risk perception in society and a higher need for orientation, and potentially increased news consumption to satisfy this need of orientation (Ball-Rokeach 1973). Hence, the first research question addressed in the current study is:

RQ1. How did the frequency of news consumption about COVID-19 change throughout different phases of the COVID-19 pandemic in the Netherlands?

Another aspect to address when unpacking news consumption and how to best reach people during times of crisis relates to the specific *news information channels* on which the crisis-related news is placed. The main categories of news information channels in the current media landscape include television, print, radio, and online (Reich 2016). Along with these media channels, it has also been suggested that the (online) interpersonal environment of a person (e.g., family, friends, neighbours, colleagues) can act as an important news information channel (Larsen and Hill 1954; Tsao et al. 2021). All these channels differ in terms of characteristics and affordances (Vonbun-Feldbauer and Matthes 2018), which can arguably impact their usage in times of crisis. For example, it has been suggested that online news information channels will be more frequently used, compared to traditional news information channels (e.g., print, radio, TV), due to their 24/7 easy and immediate access wherever someone is (van Aelst et al. 2021).

On the other hand, traditional news information channels may be perceived as more credible and less likely to publish mis- or disinformation, which can lead to a preference towards them, particularly during a crisis when the need for accurate information is high (Newman et al. 2021; van der Meer 2018). Higher usage of specific news information channels during crises is also possibly impacted by whether the news information channel provides information with expertise on the crisis (e.g., medical sources during health crises; Boyce 2006) or authority related to the management of the crisis (e.g., governmental sources during national-wide crises; Tsang, Zhao, and Chen 2021). Hence, to provide insights into the channels that best reach different population segments during a pandemic, the second research question of this paper is:

RQ2. What news information channels were used in different stages of the COVID-19 pandemic in the Netherlands?

Third, it is important to consider the news consumer characteristics that may be influencing news consumption throughout a long-term crisis (Falcone and Sapienza 2020; Sulistyawati et al. 2021; Westlund and Ghersetti 2015). For example, traditionally, men have been more frequent consumers of news media compared to women (Benesch 2012; Sindermann et al. 2020), also in the Dutch context (Commissariaat voor de Media 2021). Similarly, older (vs. younger) people have been generally shown to consume news more frequently—with a preference for traditional news channels like TV and print media vs. online media (Boulianne and Shehata 2022; Commissariaat voor de Media 2021). However, little is known about whether these differences hold during different phases of a pandemic as the news consumption gaps could theoretically both diminish or amplify. For example, the gender gap in news consumption may change during a crisis like the COVID-19 pandemic, with especially women found to increase their news consumption at the beginning of the pandemic (Ladis, Gao, and Scullin 2023; Lemenager et al. 2021). Also, certain age groups may become more active news consumers compared to before the pandemic, for example due to a substantial increase in the time spent at home (Broersma and Swart 2022); or if, on the contrary, some age groups tend to even more frequently avoid the news, for example due to negative effects of the pandemic on their mental health (De Bruin et al. 2021).

Furthermore, education has been shown to be a predictor of news consumption (Edgerly 2022; Merten et al. 2022; Shehata and Strömbäck 2011), with a potentially increased news consumption gap during the pandemic, due to, for example, more opportunities to work from home and access news media for people with a higher (vs. lower) education level. Likely, migration background is also potentially related to news consumption frequency during crises (Christiansen 2004), considering factors that might hamper media consumption, such as language barriers, time to consume media, and access to paid-for media channels, or, in the context of COVID-19, more consumption due to the increased need for information regarding the unfolding of the crisis in other countries where family members reside.

Although, to our knowledge, it has not been studied yet, the health vulnerability towards the COVID-19 illness is likely also related to the frequency of news consumption, due to a higher perceived risk of the virus which makes the topic more salient in these groups. Similarly, someone’s living situation (e.g., alone or together with someone), and working situation (e.g., being employed or freelancer) may influence the frequency of news consumption on the topic of the pandemic, given the variation of the impact of regulations on social distancing and closure of certain businesses (World Bank 2022).

Another possible predictor of news consumption during a crisis is the trust in the entity primarily responsible for managing a crisis, in the context of COVID-19: the government. Governmental authorities have been commonly used as journalistic sources in news about COVID-19 (Bernaola-Serrano and Aguado-Guadalupe 2022); hence, the trust (or lack thereof) in these authorities may be related to the perceived credibility and usage of certain news information channels and news in general**.** Previous research has studied the relationship between news media consumption and trust in government (or political trust more broadly) by focusing particularly on the role of media consumption as a predictor of political trust, rather than the other way around (Avery 2009). Theories diverge on whether this relationship is positive (virtuous circle theory, e.g., Norris 2000) or negative (media malaise theory, e.g., Robinson 1976). A study by Strömbäck, Djerf-Pierre, and Shehata (2016) showed furthermore that the relationship between media use and political trust varies among different media types (e.g., newspapers, public service TV, or commercial TV news). Given these nuances, and because news media consumption may fluctuate during relatively short periods of time throughout a crisis, it is therefore relevant to explore the possible predicting role of trust in government on news media consumption during the COVID-19 pandemic; especially, because in that time politics (and political decisions) had such a direct impact on citizens’ lives. From a pandemic preparedness perspective, trust in government is associated with higher adherence to behavioural regulations during the pandemic (Gratz et al. 2021), thus it is important to assess via which news information channels people with a higher or lower trust in government can be best reached. Nevertheless, as far as we are aware, this has not been explored yet in the context of COVID-19.

Hence, our third and final research question focuses on the impact of the abovementioned socio-demographic characteristics and trust in government on news consumption about COVID-19 in general, as well as on the usage of different news information channels about COVID-19 in different stages of the pandemic:

RQ3. Who were the more frequent users of (a) news about COVID-19 in general and (b) news information channels about COVID-19 in particular, in terms of their age, gender identity, education, migration background, health vulnerability, work situation, living situation, and trust in government throughout the different stages of the COVID-19 pandemic in the Netherlands?

## Methods

### Data and Study Design

Data were collected as part of a longitudinal nationwide cohort study conducted in the Netherlands during the COVID-19 pandemic between 17 April 2020 and 11 September 2022**.** The study was carried out by the Behavioural Unit of the National Institute for Public Health and the Environment (RIVM) in the format of an online survey in the Dutch language**.** There were 21 rounds of data collection (see Appendix 1 for the exact periods and corresponding policy regulations). Data on general news consumption and usage of different news information channels were measured in 18 and 9 rounds, respectively. The complete questionnaires are openly available (Nationaal Georegister 2024). The final sample size included in our analyses consisted of 83,180 unique respondents, offering a total of 306,692 unique responses. A more detailed description of the cohort is available elsewhere (van den Boom et al. 2023).

### Measurements

***News media consumption*** was measured with the item “In the past 7 days (1 week), did you follow the news about the coronavirus?” (0 = *never* to 1 = *always*).

***News information channels*** were measured with the item “Which sources were important for you for news and information about the coronavirus in the previous 7 days? (more answers are possible)” (per news information channel, 0 = *no*, 1 = *yes*), followed by a list of 16 types of news information channels, drafted by a group of experts and communication scholars in the Netherlands (see Appendix 2). An exploratory principal component analysis (PCA) was run, in order to assess whether dimension reduction could be applied, resulting in five factors with an eigenvalue higher than 1, explaining 53.41% of the variance of the 16 news information channels. These main groups were labelled as (1) *TV news*; (2) *newspapers and local media*; (3) *(online) interpersonal communication;* (4) *governmental channels,* and (5) *medical channels*. Two items did not load sufficiently on any of the five factors and therefore, for RQ3, were analysed separately, namely (6) *online news* and (7) *radio*. The factor loadings and corresponding items for each news information channel are shown in Table 1. The factors were transformed in new variables, with the values “0” if none of the corresponding news information channels were used by the respondents, and “1” if at least one of the corresponding news information channels was used by the respondents.

**Table 1.** Corresponding items and eigenvalues of the identified groups of news information sources.

News information channel factor	TV news	Newspapers and local media	(Online) Interpersonal communication	Governmental channels	Medical channels	Online news website	Radio
Eigenvalue	2.39	1.05	1.22	1.77	1.08	NA	NA
Items (and corresponding factor loadings)	– News bulletins on public broadcasting channels, such as NOS journal (.69) – News bulletins on commercial broadcasting channels, such as *RTL nieuws* (.63) – News background programs such as *Nieuwsuur* or *EenVandaag* (.75) – Talkshows such as *Op1* or *De Vooravond* (.73)	– National newspapers (.84) – Regional and local media (.48)	– Social media, such as Facebook, Twitter, or Instagram (.77) – People in my environment, such as neighbours, colleagues, or family (.72)	– Website of the Dutch government (.71) – Website of the RIVM (.74) – Website of my regional GGD (.61) – Website of the municipality where I live (.49)	– General Practitioner (.77) – Medical websites, such as thuisarts.nl (.74)	– Online news websites or apps, such as nos.nl or nu.nl	– Radio channels

***Age*** was measured with six categories (0 = *16–24 years*, 1 = *25–39*, 2 = *40–54*, 3 = *55–69,* 4 = *70–84,* 5 = *85+*).

***Gender identity*** was measured with three categories (0 = *woman*, 1 = *man*, 2 = *other*). Due to the very small percentage of respondents who identified as “other” (< 0.1%), this category was not included in the multilevel analyses.

***Education level*** was measured with three categories (0 = *low*, 1 = *medium*, 2 = *high*).

***Living situation*** was measured with two categories (0 = *living alone*, 1 = *living together with someone*).

***Work situation*** was measured with two separate items, namely *being employed* “Are you currently employed with an employment contract?” (0 = *no*, 1 = *yes*) and *being self-employed “*Are you currently a freelancer or business owner?” (0 = *no*, 1 = *yes).*

***Health vulnerability*** was measured with the item “Do you have one or more of the following health problems” (per health problem, 0 = *no*, 1 = *yes*). A list of health problems associated with a higher health vulnerability related to COVID-19 was included in the answer options**,** based on the expertise of public health professionals involved in design of the questionnaire**:** chronical respiratory problems, chronical heart problems, diabetes, lower immunity against infections, and risky BMI. The final variable was recoded into 0*,* if no health problem was mentioned and 1, if the respondent had at least one health problem.

***Migration background*** was measured with the item “In which country were you born?” (eight categories, 1 =  *The Netherlands*, 2 = *Dutch Antilles/Aruba*, 3 = *Dutch East Indies/Indonesia*, 4 = *Morocco*, 4 = *Suriname*, 6 = *Turkey*, 7 = *Somewhere else in Europe/North America*, 8 = *Somewhere else outside Europe/North America*). Answer option 1 was coded as “no migration background”, 2–6 and 8 as “non-western migration background” and 7 as “western migration background” (CBS 2023). Subsequently, these two categories (i.e., non-western and western) were recoded as dummy variables, in order to be included in the statistical analyses (see Appendix 3 for the percentage of respondents in each category).

***Trust in government*,** measured with the scale item “How much trust do you have in the way the Dutch government tries to keep the coronavirus under control?” (0 = *no trust at all*, to 4 = *very much trust;* M = 3.37; SD = .90).

### Analyses

The initial analysis plan of the current paper, including the research questions, has been preregistered on OSF (Solovei et al. 2022). In addition to the initial analysis plan, more advanced multilevel regression tests were performed as described below. To answer RQ1 and RQ2, we conducted descriptive analyses of each variable. To answer RQ3a, a multilevel linear regression was run, with the available rounds of data collection included as a random effect in the model, news media consumption as the dependent variable and socio-demographic characteristics, trust in government, and time (operationalized as the round number) as the predictors—included as fixed effects in the model. To detect any potential time trends of the effects of the abovementioned predictors, interaction terms between each predictor and time were included additionally as fixed effects, in a separate model. To answer RQ3b, seven multilevel binary logistic regressions were run, with the respondents nested under the rounds of data collection (random effect), each news information channel factor identified in RQ2 as the dependent variable and socio-demographic characteristics, trust in government, and time as the predictors (fixed effects). Interaction terms between time and each socio-demographic predictor were added in a separate model. Given the large sample size, which increases the likelihood of finding statistically significant results even if the effect sizes are not substantial, we preselected a threshold of B > 0.10 (for the linear regression) and odds ratio OR >1.40 (or alternatively, OR <.70) as what can be considered an effect size worth considering in the interpretation of results (Cohen 1988). Additionally, for a clearer picture of the specific patterns of news media consumption amongst the different socio-demographic groups, we analysed the marginal means pertaining to the general news consumption and the different news information channels, for those socio-demographic factors that were revealed to be substantially impacting the dependent variables.

#### Robustness Analyses

To further assess whether the results pertaining to RQ3 remain similar when tested on a stable sample of respondents we conducted robustness analyses, using a complete case analysis approach. For feasibility reasons, the analyses were done on five preselected rounds, namely rounds 1, 5, 8, 10, 19. This resulted in a sample of 4786 participants who participated in all 5 rounds of data collection and completed the relevant items included in our analyses (see Appendix 4 for socio-demographic characteristics). The five rounds were selected by the researchers to represent the periods in which the most important policies for containing the COVID-19 pandemic were implemented, either in terms of strict(er) regulations (three rounds) or in terms of relaxation of regulations (two rounds). For each of these five rounds linear regressions were run, with news media consumption and usage (yes/no) of the news information channels as the dependent variables and socio-demographic characteristics and trust in government as the predictors.

## Results

### Frequency of News Consumption About COVID-19

**Figure 1.**
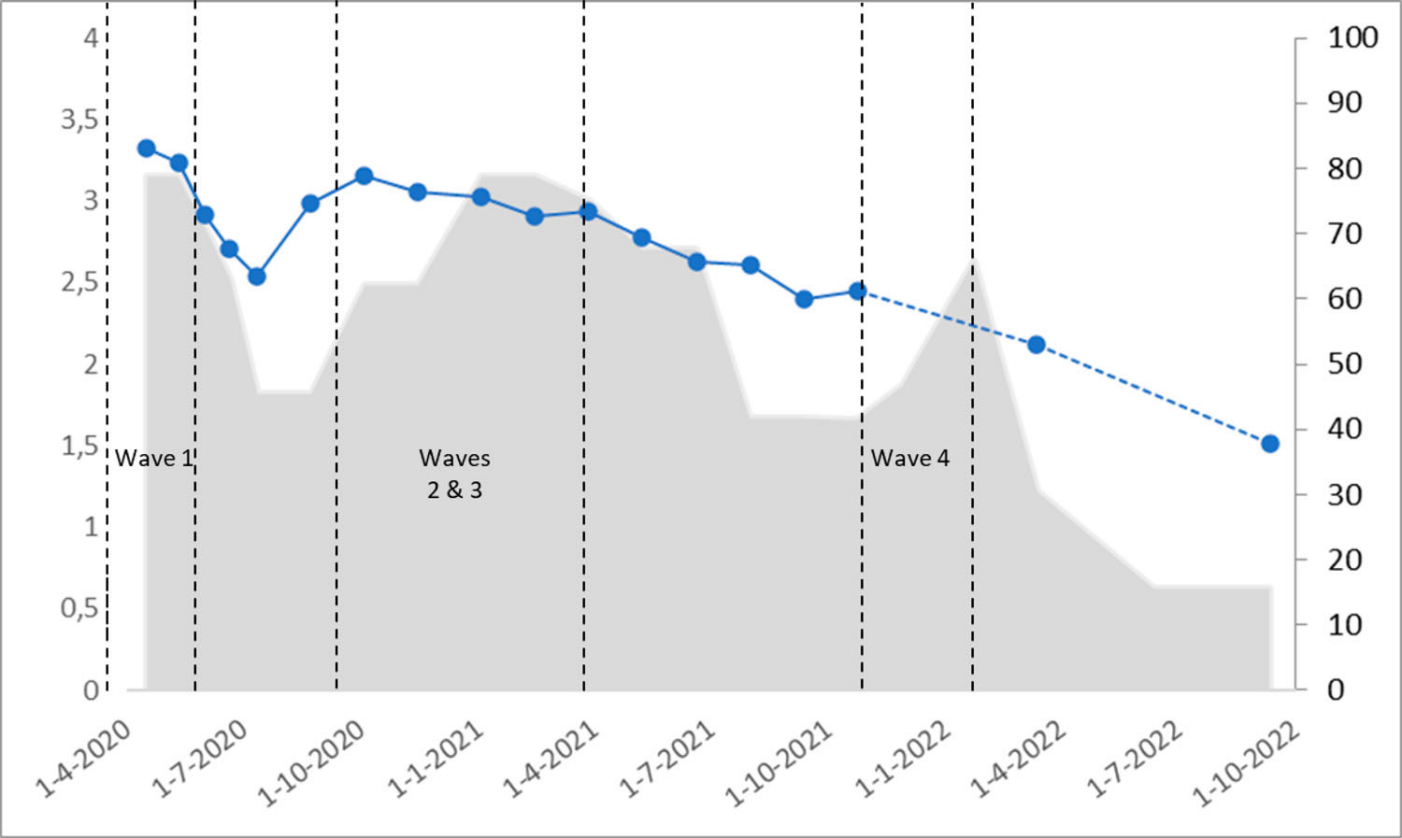
Frequency of news consumption about COVID-19 across the different data collection rounds and pandemic stringency index*.* Note. News consumption frequency (blue line, corresponding Y-axis shown on the left of the chart) was measured on a scale from 0 (never) to 4 (always). Data on this variable were not collected in Round 17, 18 and 20, which is reflected by a dashed blue line in the chart. The pandemic stringency index (gray background, corresponding Y scale shown on the right of the chart) was measured on a scale from 0 (no restrictions) to 100 (most stringent restrictions). Both news consumption frequency and pandemic stringency index are shown for the previous 7 days of the start of each respective measurement round. N = 306,692 responses, pertaining to 83,180 unique respondents. The phases marked with dashed vertical lines and labelled as “wave” indicate the waves of infections during the pandemic in the Netherlands.

RQ1 focused on the frequency of COVID-19-related news consumption throughout the COVID-19 pandemic. As illustrated in Figure 1, news consumption was at its highest at the beginning of the pandemic, decreased in the summer of 2020 (when relaxed regulations were in place), increased again in the fall of 2020 (when there was another surge of infections and stricter regulations were announced), and followed a gradually decreasing trend afterwards.

### Usage of Different News Information Channels

**Figure 2.**
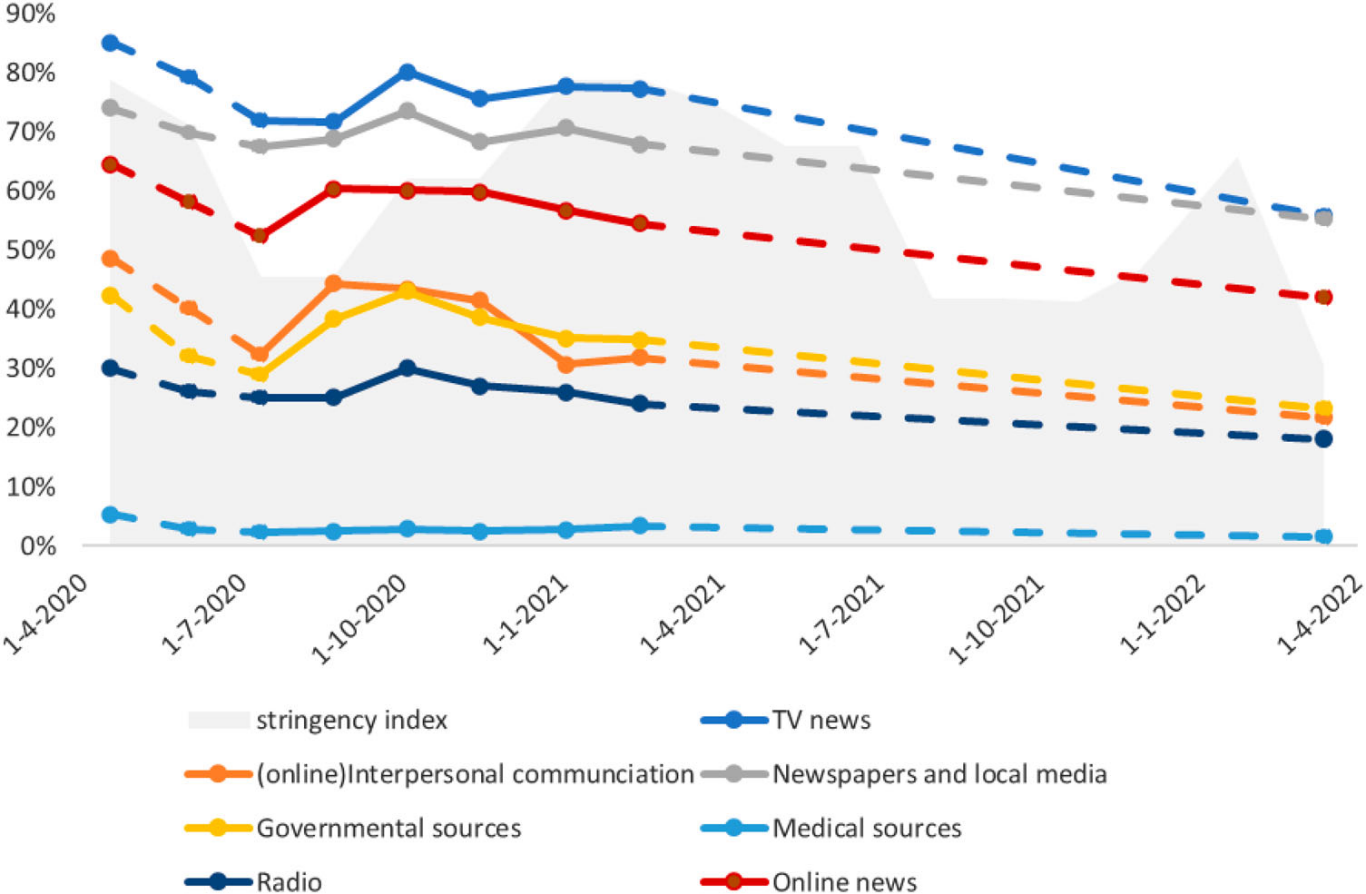
Percentage of respondents using different news information channels in the different rounds of data collection*.* Note. No data available for Round 2, 4,11-18, 20 and 21, indicated by the dashed lines. For presentation parsimony reasons, a singular Y-scale is used for both the percentage of respondents and pandemic stringency index.

RQ2 focused on what news information channels were used in different stages of the COVID-19 pandemic in the Netherlands. Figure 2 shows the percentage of respondents using each type of news information channel, indicating that the usage of all news information channels was at its highest at the beginning of the pandemic, and generally decreased afterwards. Notably, the usage of (online) interpersonal communication, online news and governmental channels had an increase at Round 6 (end August 2020), shortly before the start of the second wave of infections in the Netherlands. Consumption of TV news, newspapers and local media, and radio news, on the other hand, remained stable at Round 6, but increased at Round 7 (beginning October 2020)—during the second wave of infections. After Round 8 (November 2020), interpersonal communication saw a relatively steep decrease in use. Throughout the whole pandemic, TV news was used by the most respondents in our sample, followed by local and national newspapers and online news. Governmental sources, radio, and (online) interpersonal communication were each used by about 30–40% of the respondents, while medical channels were used by less than 5% of the respondents, on average.

### Socio-demographics Predictors of News Consumption and News Information Channels

RQ3 aimed to explore the socio-demographic factors impacting (a) the general news consumption about COVID-19 and (b) the usage of different news information channels; throughout the COVID-19 pandemic. All results are summarized in Tables 2 and 3, both including two models for each dependent variable: Model 1 contains only the main effects of the socio-demographic predictors and time, whereas Model 2 also includes the interaction effects between time and each socio-demographic predictor. Unless otherwise specified, the results were similar between these two models for each dependent variable.

**Table 2.** Socio-demographic predictors of general news consumption and usage of tv news, newspapers, online news and radio.

	TV news		Newspapers		Online news		Radio	
	Model 1	Model 2	Model 1	Model 2	Model 1	Model 2	Model 1	Model 2
Intercept	5.51***	5.87***	3.09***	3.18***	1.51***	1.48***	.37***	.38***
**Main effects**								
Gender (male)	1.12***	1.03	**1**.41***	1.33***	1.20***	1.21***	1.23***	1.17***
Age	**1**.70**	**1**.89***	**1**.49***	**1**.48***	.70***	.70***	1.10***	1.12***
Education level	1.07***	1.09***	1.22***	1.22***	1.31***	1.32***	1.04***	1.01
Living situation (alone)	.85**	.75***	.82***	.77***	.88***	.82***	1.06***	1.02
Work situation (employed)	.87***	.87***	.85***	.87***	1.21***	1.25***	1.03	1.01
Work situation (freelancer)	.78***	.76***	.95**	.94	1.09***	1.13***	1.06*	1.03
Health vulnerability (yes)	1.08***	1.12***	.96**	.97	.99	1.01	.91***	.90
Migration background (western)	.56***	.47***	.71***	.74***	.91*	.88	1.00	1.11
Migration background (not western)	.69***	.61***	.62***	.55***	.82***	.80**	.75***	.79
Trust in government	**1**.41***	**1**.42***	1.18***	1.15***	1.16***	1.20	1.14***	1.11***
Time	.94**	.99	.96***	.96***	.96***	.98	.97***	.97***
**Interaction effects**								
Gender * time		1.01***		1.01***		1.00		1.00**
Age * time		.99***		1.00**		1.00		1.00
Education * time		1.00		1.00		1.00		1.01***
Living situation * time		1.02***		1.01**		1.01**		1.01
Work situation (being employed) * time		1.00		1.00		1.00		1.00
Work situation (being self-employed) * time		1.05		1.00		.99		1.00
Health vulnerability * time		1.00		1.00		1.00		1.00
Migration background (western * time)		1.03***		.993		1.01		.98
Migration background (not western * time)		1.03***		1.01		1.00		.99
Trust * time		1.00		1.00**		1.00***		1.00**
AIC	524569.49	525851,74	493282,21	492771.55	479651.75	479285.45	498009.04	497682.12
*R*^2^ marginal	.11	.11	.08	.08	.08	.08	.02	.02
*R*^2^ conditional	.12	.12	.08	.08	.09	.09	.02	.02
*N*	110274	110153	110274	110153	110274	110153	110274	110153
ICC	.01	.01	.00	.00	.01	.01	.00	.00

Note*.* Substantial B-values (>.10) and OR-values (either < .72 or >1.40) are emphasized in bold; *p < .05; **p < .01; ***p < .001. Model 1 refers to models including main effects only, Model 2 refers to models including both main and interaction effects.

**Table 3.** Socio-demographic predictors of general news consumption and usage of governmental channels, medical channels, and interpersonal communication.

	General news		Governmental channels		Medical channels		(online) Interpersonal communication	
	Model 1	Model 2	Model 1	Model 2	Model 1	Model 2	Model 1	Model 2
Intercept	2.53***	2.72***	.65**	.64***	.05***	.05***	1.07	1.07
**Main effects**								
Gender (male)	.07***	-.01	.83***	.80***	1.08*	.90*	.92***	.86***
Age	.15***	.13***	.85***	.80***	.93***	.90***	.69***	.67***
Education level	.07***	.02***	1.08***	1.06***	1.07***	1.13***	.96***	.98**
Living situation (alone)	**-**.11***	**-**.14***	.93***	.96	1.00	.97	1.05**	1.01
Work situation (employed)	-.05***	-.01	1.13***	1.20***	.80***	.87*	1.05***	1.12***
Work situation (freelancer)	-.05***	-.05***	1.20***	1.30***	1.00	1.08	1.08***	1.15***
Health vulnerability (yes)	.07***	.06***	1.22***	1.21***	**1**.83***	**1**.88***	1.03**	1.03
Migration background (western)	.07***	.05*	**1**.55***	**1**.47***	1.08	.93	1.05	1.07
Migration background (not western)	.07***	.09***	1.13*	1.26***	1.28**	1.12	1.05	1.08
Trust in government	.16***	.15***	1.17***	1.11***	.95**	.95*	.93***	.94***
Time	-.06***	-.08***	.98	.94***	.94***	1.00*	.93***	.95***
**Interaction effects**								
Gender * time		.01***		.99*		1.03***		.99***
Age * time		.01***		1.01***		1.01		1.00*
Education * time		.01***		1.00*		.99**		1.00*
Living situation * time		.01***		1.00		1.01		1.01
Work situation (being employed) * time		-.01***		.99***		.98		.99**
Work situation (being self-employed) * time		.01		.99***		.99		.99**
Health vulnerability * time		.01		1.00		1.00		1.00
Migration background (western * time)		.01		1.01		1.03		1.00
Migration background (not western * time)		-.01*		.99		1.02		.99
Trust * time		.01***		1.01		1.00		.99
AIC	594281.90	593891.84	479804.91	479568.23	696770.79	698513.78	481815.79	481135.33
*R*^2^ marginal	.21	.21	.03	.03	.01	.01	.07	.07
*R*^2^ conditional	.27	.27	.05	.05	.01	.01	.09	.09
*N*	306692	306692	110274	110153	110274	110153	110274	110153
ICC	.07	.07	.01	.02	.00	.00	.02	.02

Note*.* Substantial B-values (>.10) and OR-values (either < .72 or >1.40) are emphasized in bold; *p < .05; **p < .01; ***p < .001. Model 1 refers to models including main effects only, Model 2 refers to models including both main and interaction effects.

**Figure 3.**
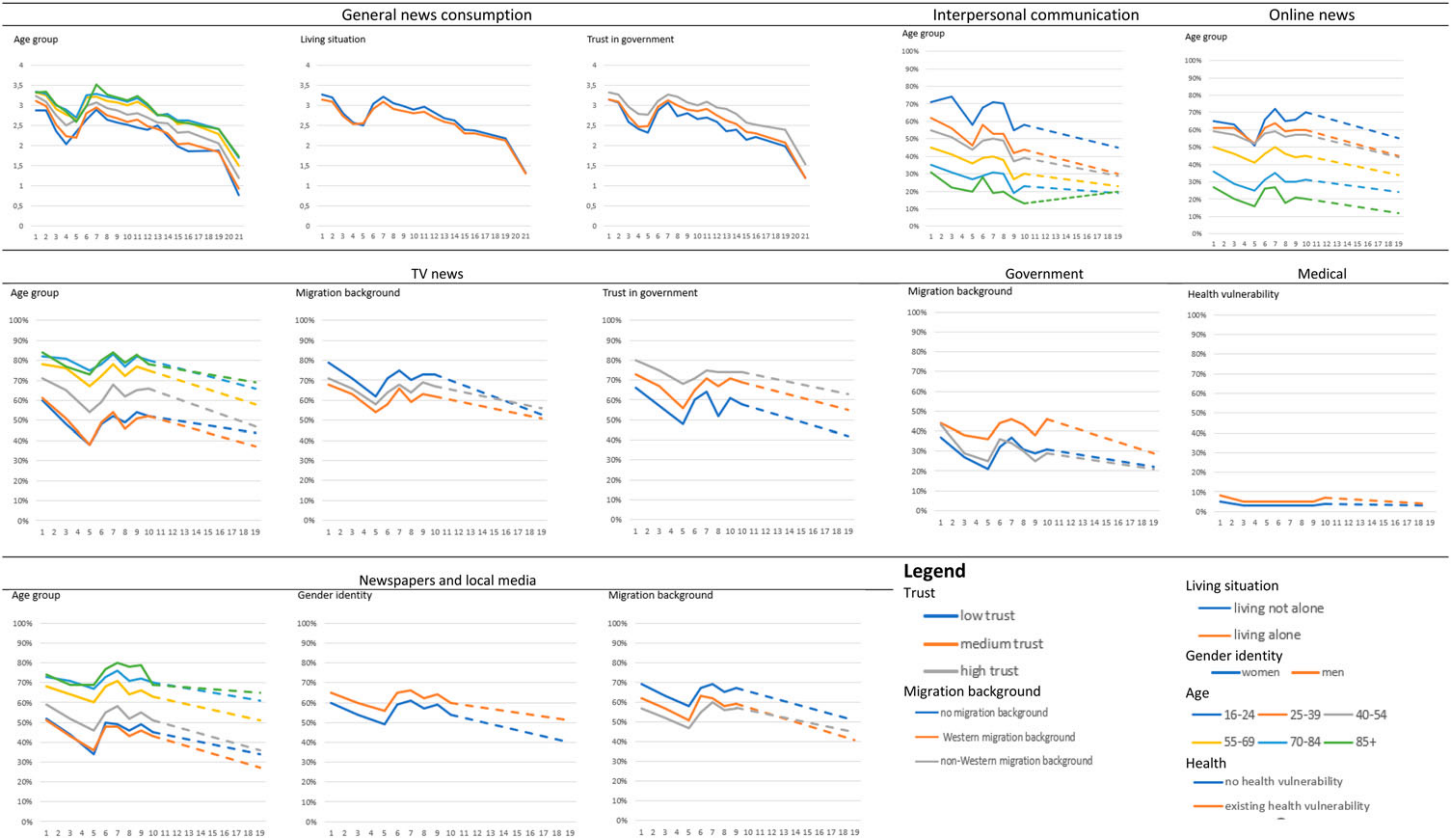
Marginal means of general news consumption and news information channel usage in different socio-demographic groups at all measurement rounds.

For RQ3a, the multilevel linear regression analysis showed that, throughout the pandemic, there were several predictors of more frequent general news consumption about COVID-19, namely having an older (vs. younger) age, not living alone (vs. living alone), and having a higher (vs. lower) trust in government. The other predictors, as well as all-time interaction effects, although for a large part statistically significant, did not have substantial effect sizes, with beta coefficients lower than the pre-established threshold of .10. For RQ3b, the multilevel logistic regression analyses revealed the following results. TV news was more likely to be used as a COVID-19-related news information channel by older (vs. younger) people, people without a migration background (vs. western or non-western migration background); and people with higher (vs. lower) trust in the government. Newspapers and local media were more likely to be used by men (vs. women, but only in model 1 without time interaction effects), older people, and people without migration background. A further investigation of the effects of gender identity on usage of newspapers and local media (see Figure 3) showed that at the beginning of the pandemic men and women consumed news about COVID-19 in newspapers and local media more similarly to each other, compared to the later stages of the pandemic—when men were more likely to use this news information channel. Governmental channels were more likely to be used by people with a western migration background (vs. without migration background). Medical channels were more likely to be used by people having a health vulnerability, while online news and (online) interpersonal communication were each more likely to be used by younger people. The likelihood of radio consumption was not substantially impacted by any of the tested socio-demographic factors.

The estimated marginal means depicted in Figure 3 show that the consumption of general news about COVID-19 followed similar decreasing time trends across all analyzed subgroups. In terms of absolute numbers, the differences between the youngest and oldest age groups were more substantial, compared to the differences based on living situation or trust in government. Moreover, the marginal mean results in Figure 3 showed similar decreasing trends amongst all included socio-demographic groups, for all news information channels. Interestingly, the results indicate that, despite the negative relationship between age and usage of TV news, and newspapers and local media, these news information channels still reached a substantial proportion of the young people in our sample, with more than half of the young respondents being consumers of TV news, and/or newspapers and local media at the beginning of the pandemic. Nevertheless, online news websites and (online) interpersonal communication were the most used news information channels amongst young respondents, reaching around 70% at the onset of the pandemic. An overview of the marginal means corresponding to the socio-demographic groups and each news information channel can be found in Appendix 5.

Furthermore, the results of the robustness analyses are presented in Appendix 6 and overall confirm the main results. A difference is that in the robustness analyses the education level had a more substantial positive association with the consumption of online news (in four of the five preselected waves), with odds ratios just above the cut-off score of 1.40, whereas in the main results, the odds ratios approached the cut-off but remained below it. This means that, people with higher education levels may have been more likely to consume news about COVID-19 via online news website, compared to people with lower education.

## Discussion

This study has explored news consumption patterns during the COVID-19 pandemic in a dynamic cohort in the Netherlands. News consumption about COVID-19 was the highest at the beginning of the pandemic in April 2020, decreased in the summer of 2020 (when policies were relaxed), increased in the fall of 2020 (when stricter regulations were announced), and followed a gradual decreasing trend afterwards, with some periods of stagnation. Furthermore, the results show that the most used news information channel in our cohort was TV news. This holds for each of the analysed socio-demographic subgroups, except for the younger than 40 years group, who, in terms of absolute numbers, preferred online news websites (Appendix 6). Shortly before the second wave of infections (end of August 2020), the usage of interpersonal communication, online news, and governmental channels increased, whereas for TV news, radio, and newspapers and local media the increase in usage came in the following measurement round (beginning of October 2020, during the second wave of infections). Our results also reveal several positive predictors of the usage of news information channels on the topic of COVID-19. Substantial factors included having an older age (for general news consumption, TV news, and newspapers and local media), having a younger age (for [online] interpersonal communication and online news), having no migration background (for TV news, and newspapers and local media), having a western migration background (for governmental channels), living together with someone (for general news consumption), having a health vulnerability (for medical channels) and having higher trust in government (for general news consumption and TV news). These results remained stable during the pandemic.

Our results imply that people with lower trust in government are less likely to follow general news about a crisis and should hence be reached with additional or alternative—for them trustworthy—sources of information about a crisis. Examples may include (tailored or targeted) communication campaigns or interpersonal communication with trusted persons, opinion leaders in their communities and online micro-influencers, which have been shown to be effective communication strategies for more difficult-to-reach population groups (Hindhede and Aagaard-Hansen 2017; Valente and Pumpuang 2007; Zhang and Zhao 2020). Furthermore, given that people with a migration background were more likely to use governmental channels to inform themselves about COVID-19 (possibly due to information needs regarding travelling restrictions to their home countries during the pandemic), it would be important to tailor or adjust the information on governmental websites for this group, for example by offering updated translated information in the languages that are frequently used by migrants in the country of residence.

A theoretical explanation for the decrease in COVID-19-related news consumption may be that, throughout the pandemic, more knowledge became available about the SARS-COV-2 virus, its health threat, and the implemented behavioural regulations, so that the public need for orientation was lower than in the beginning of the pandemic, when more uncertainty was at play. This is in line with the media systems dependency theory (Ball-Rokeach 1985), which posits that news consumption is expected to increase in times of greater uncertainty but decreases when there is less uncertainty.

Our results also indicate that traditional media, like TV and newspapers, remain important news information channels for acquiring information about a crisis. This implies that using traditional media to disseminate crisis information, at least at the starting phases of a crisis, may be recommendable to reach large proportions of the public. Analyses from the Digital News Report in the Netherlands indeed confirm that general news consumption had a remarkable spike during the beginning of the COVID-19 pandemic, compared to the year before, including a substantial increase of users of paid news (Commissariaat voor de Media 2021). While trust in government in the Netherlands decreased during the crisis (from up to 70% of the population at the start of the crisis to around 30% towards its end; RIVM 2021), trust in news remained relatively high and stable, with 59% of the population trusting the news in 2021 (top 3 in Europe). The public broadcaster NOS had the highest trust scores among both users and non-users (7.8 and 7.4 out of 10, respectively). These patterns of high trust in news also apply to other mainstream news media channels investigated in our study, which indicates relatively low polarization in the Netherlands with regards to trust in the mainstream media. It is likely that longitudinal patterns of news consumption in a different political, sociocultural and/or media context would have produced different results. Hence, our findings may be less applicable to contexts characterized by lower trust in news and higher polarization (Kritzinger et al. 2021).

The noticed delay in increased usage of TV, radio and print news shortly before the start of the second wave of infections (in contrast to interpersonal communication or online news) aligns with the findings of Groot Kormelink and Klein Gunnewiek (2022). Their study of the first COVID-19 wave in the Netherlands also identified an initial spike in news consumption at the beginning of the pandemic, followed by an avoidance phase in which consumers reduced their engagement with traditional news. This pattern was followed by a normalization phase, where news consumption increased again, similar to the “regained stability” phase described by Moe, Nærland, and Ytre-Arne (2023). In addition, a study in Flanders on young news consumers (Vandenplas et al. 2021) found that many reduced their news intake during the COVID-19 pandemic, rather than avoiding it altogether. This behaviour seems to be reflected in our study where people reduced their traditional news consumption in the summer of 2020 and resumed it in the autumn as their news habits normalized.

The overall decreasing trend in the usage of the various news information channels observed in our study throughout the COVID-19 pandemic also implies that the public might gradually experience a less stringent need for information during the unfolding of a crisis. These findings offer general support for the sequential typology proposed by Moe, Nærland, and Ytre-Arne (2023), which predicted such a stage of regained stability. Nevertheless, as mentioned above, our results also showed that, at least in the first pandemic year, news consumption can fluctuate at points in time when the crisis becomes more serious again (e.g., at the start of new waves of infections). This can be explained by the “regenerative” character of the crisis (Mak and Song 2019), entailing that within a large-scale crisis, sub-crises can keep occurring at certain points in time, therefore leading to fluctuations in crisis-related news consumption: the state of “shocked immersion” may occur multiple times in the same crisis.

Overall, our results reveal that different demographic groups can vary in their news usage during crises in terms of frequency and channels, highlighting the importance of in-depth future research regarding the motivations, needs, and habits that underlie patterns of news consumption of different socio-demographic groups and across different channels. This would contribute to a better understanding of the circumstances and mechanisms that explain how uses and gratifications theory best applies in times of crisis. For example, one could study during a crisis which needs are the most prevalent and important in different phases, amongst different socio-demographic segments, and how these could be best gratified through different news information channels. Additionally, in future research, contextual factors could be included as explanatory variables (e.g., quantifications of the crisis context, such as the number of infections) to better understand how news consumption during crises is influenced by real-world developments.

In terms of implications for pandemic preparedness research, it seems that news items about a crisis will likely receive less attention in later phases of that crisis. For example, between Rounds 16–19 the number of daily occupied COVID-19 hospital beds increased from 430 to 1330 (Appendix 1), but our data showed that news consumption about COVID-19 decreased in that same period, suggesting the occurrence of the third sequential phase proposed by Moe, Nærland, and Ytre-Arne (2023), known as “regained stability”. So, in the case that (new) behavioural regulations are required from the population, both policymakers and journalists could benefit from knowledge regarding how to attract the attention of all members of the public to convey the crisis-related news. Journalists could consider increasing the coverage (e.g., number of news items) regarding the topic, however this strategy might boomerang within sections of the public who are more inclined to avoid the news on the subject (Groot Kormelink and Klein Gunnewiek 2022). Moreover, for policymakers, it is advisable to consider alternative channels for information dissemination, in addition to news information channels (e.g., public information campaigns, dashboards, (online) community meetings) (Vahedi et al. 2022; Weiss and Tschirhart 1994).

### Strengths and Limitations

The main strengths of this study include the large cohort sample of respondents, with 18 rounds of data collection, spanning over 2.5 years of the COVID-19 pandemic. This makes it, to the best of our knowledge, one of the first studies covering longitudinal patterns of news consumption throughout the long-term COVID-19 pandemic (up until September 2022, by when most pandemic regulations were given up worldwide). Nevertheless, certain limitations need to be considered when interpreting the results. First, in spite of its large size, the sample is not a representative sample of the Netherlands and it is likely that it had a higher interest in the COVID-19 topic, compared to the rest of the Dutch population. Also, the respondents with a migration background were all able to read and understand Dutch. Nonetheless, our sample format did allow detecting longitudinal patterns of news consumption, which were additionally confirmed by our robustness analyses in a complete case sample. Taking the abovementioned limitations into account, future research, particularly in the context of crisis communication and pandemic preparedness, could benefit from using a more representative panel of respondents who participate in multiple rounds of data collection, before and during a (health) crisis.

To conclude, our paper showed that the news consumption varied in different phases of the COVID-19 pandemic in the Netherlands, with an overall decreasing trend in news consumption throughout the crisis. The most commonly used news information channels were TV news bulletins, newspapers and local media, and online news. Moreover, age, migration background, and trust in government were common predictors of the use of different news information channels during the COVID-19 crisis.

## Supplementary Material

Apppendices

## Data Availability

Due to European privacy regulation restrictions (GDPR) and the nature of the research, the data cannot be shared publicly unless aggregated.
