# Supplementary material for: Patterns of News Consumption during the COVID-19 Pandemic Crisis: A 2.5 Year Longitudinal Study in the Netherlands
Source: Journal Stud. 2024 Sep 27;25(16):1968–89. doi: 10.1080/1461670X.2024.2407944 (PMC11601047; doi:10.1080/1461670X.2024.2407944)
Supplement: Apppendices [file RJOS_A_2407944_SM1232.doc]

**Appendices**

**Appendix 1**

*Data collection rounds and corresponding periods*

| **Round*** | **Period of data collection** | **Main COVID-19 regulations**** | **Pandemic stringency index***** | **Average number of daily occupied COVID-19 hospital beds****** |
| --- | --- | --- | --- | --- |
| **1** | 17– 24 April 2020 | - 1.5 meter distancing in all public spaces;  - Washing hands regularly;  - Avoiding shaking hands;  - Avoiding visiting vulnerable groups (e.g., older people, people with health conditions);  - Working from home, except for essential jobs;  - Closure of schools, child care centres, restaurants and cafés, places with contact professions (e.g., hair salons);  - Lockdown imposed on March 23. | 79 | 2530 |
| 2 | 7 – 12 May 2020 |  | 79 | 1410 |
| **3** | 27 May – 1 June 2020 |  | 71 | 810 |
| 4 | 17 – 21 June 2020 | - Relaxed regulations  - Lockdown ended on June 1.  - Restaurants and cafés allowed to open with restrictions | 63 | 310 |
| **5** | 8 – 12 July 2020 | - Further relaxed regulations:  - No limit of number of people that can be together in public spaces, on the condition that a 1.5-meter distance is kept between all individuals (besides children <18). | 45 | 110 |
| **6** | 19 – 23 August 2020 | - Stricter regulations;  - Restriction on the number of people that one can receive at home daily (no more than 6). | 45 | 120 |
| **7** | 30 September – 4 October 2020 | - Stricter regulations;  - Restriction on the number of people that one can receive at home daily (no more than 3). | 62 | 460 |
| **8** | 11 – 15 November 2020 | - Stricter regulations;  - Restriction on the number of people that one can receive at home daily (no more than 2).  - Restaurants and cafés  - Public events prohibited;  - Sport activities of max. 2 people, group sport activities prohibited;  - General advice to stay as much as possible at home. | 62 | 1800 |
| **9** | 30 December 2020 - 3 January 2021 | - Stricter regulations;  - Lockdown imposed on December 15;  - Wearing masks in all public spaces;  - Shops closed. | 79 | 1880 |
| **10** | 10 - 14 February 2021 | - Stricter regulations;  - Curfew from 21.00 – 4.30 am;  - People can receive a max. of one visitor (>13) at home;  - Schools, child care centres, restaurants, cafés, shops closed.  - On January 8, vaccination campaign started. | 79 | 1490 |
| 11 | 24 – 28 March 2021 | - Somewhat relaxed regulations;  - Shops can open, but only for customers with appointments;  - Young people (< 26) are allowed to sport in groups outside. | 75 | 1500 |
| 12 | 5 – 9 May 2021 | - More relaxed regulations;  - Curfew is ended;  - Higher education, terraces, shops open with restrictions;  - Max 2 guests older than 13 daily. | 68 | 1890 |
| 13 | 16 – 20 June 2021 | - More relaxed regulations;  - Restaurants and cafés, theatres, museums, cinemas allowed to open with restrictions;  - Team sport activities allowed outside, up to 50 people;  - For other activities, max. 4 people together in public spaces. | 68 | 460 |
| 14 | 28 July – 1 August 2021 | - More relaxed regulations since June 26;  - Most public spaces are allowed to be open, with the rule of keeping 1.5 meter distance;  - Clubs and discos are allowed to be open, with the Corona Pass;  - Work from office allowed for a max. of half of the workweek;  - Shortly afterwards, on July 5, stricter regulations are reimposed;  - Fixed seats in restaurants, cafes and public events;  - Clubs and discos closed again. | 42 | 390 |
| 15 | 8 – 12 September 2021 | - Travelling to certain countries only possible with the Corona Pass;  - On campus higher educations allowed with restrictions. | 42 | 450 |
| 16 | 20 – 24 October 2021 | - Access to restaurants, cafes and public events with the Corona Pass;  - 1.5 meter social distancing rule not mandatory anymore, but recommended;  - Work from the office allowed, if necessary. | 41 | 430 |
| 17 | 24 – 28 November 2021 | - Stricter regulations;  - Shops, restaurants and cafes open with restrictions and need to close at 5pm;  - 1.5 social distancing rule imposed;  - Max. 4 visitors at home, daily;  - Facemasks required in schools for pupils and teachers. | 47 | 1860 |
| 18 | 1. – 23 January 2022 | - On December 19, lockdown imposed, most public venues, schools, child care centres closed;  - Starting January 15 2022, more relaxed regulations;  - Nonessential shops, educational institutions, and sport clubs reopen. | 66 | 970 |
| **19** | 9 – 13 March 2022 | -Relaxed regulations:  - Almost all restrictions are given up;  - Face masks only mandatory in public transport and airports;  - Access to events of more than 500 people only allowed based on COVID-19 negative tests | 31 | 1330 |
| 20 | 8 – 12 June 2022 | -More relaxed regulations  -Face masks no longer mandatory in public transport and airports;  -Corona Pass is no longer needed for access to public venues. | 16 | 340 |
| 21 | 7 – 11 September 2022 |  | 16 | 460 |

*Note*. *No data on general news consumption were collected in rounds 17, 18, and 20. No data on usage of the different news information channels were collected in rounds 2, 4, 11-18, and 20. The rounds with complete data collection included in the analyses are emphasized in bold. ** The main COVID-19 regulation at place in the weeks preceding the start of the data collection round. ***Pandemic stringency index measured from 0 to 100, with 100 indicating the highest stringency. ****Average number of daily occupied hospital beds for COVID-19 cases in the seven days preceding the start of the data collection round.

**Appendix 2**

News information channels included in the survey

|  | **News information channel** |
| --- | --- |
|  | Website of the Dutch government |
|  | Website of the *RIVM* |
|  | Website of my regional *GGD* |
|  | Website of the municipality where I live |
|  | General practitioner |
|  | Medical websites, such as thuisarts.nl |
|  | National newspapers |
|  | Regional and local media |
|  | Online news websites or apps, such as nos.nl or nu.nl |
|  | Social media, such as *Facebook*, *Twitter*, or *Instagram* |
|  | People in my environment, such as neighbours, colleagues, or family |
|  | News bulletins on public broadcasting channels, such as *NOS Journaal* |
|  | News bulletins on commercial broadcasting channels, such as *RTL Nieuws* |
|  | News background programmes, such as *Nieuwsuur* or *EenVandaag* |
|  | Talkshows, such as *Op1* or *De* *Vooravond* |
|  | Radio |

**Appendix 3**

Socio-demographic characteristics of the respondents

| **Characteristic** | **Category** | **Percentage of responses** |
| --- | --- | --- |
| Gender identity | woman  man  other | 62.8%  37.2%  .1% |
| Age | 16-24 years  25-39 years  40-54 years  55-69 years  70-84 years  85 years or older | 2.2%  15.3 %  27.5%  35.1%  19.3%  .7% |
| Education | lower education  medium education  higher education | 12.0%  27.5%  60.5% |
| Migration background | western  non-western  no migration background | 2.4%  1.9%  95.7% |
| Living situation | living alone  living together with someone | 19.5%  80.5% |
| Work situation (being employed) | being employed with contract  not being employed with contract | 49.3%  50.7% |
| Work situation (being self-employed) | being self-employed  not being self-employed | 11.0%  89.0% |
| Having a health vulnerability | yes  no | 24.7 %  75.3 % |

*Note.* For presentation purposes, the samples characteristics are shown based on unique responses in the whole sample (N = 306,692 responses, pertaining to 83,180 unique respondents).

**Appendix 4**

Socio-demographic characteristics of the respondents in the robustness analyses

| **Characteristic** | **Category** | **Percentage of respondents** |
| --- | --- | --- |
| Age | 16-39 years  40-54 years  55-69 years  70-84 years  85 years or older | 6.8%;  21.5%  44.9%  25.8%  0.8% |
| Gender | women;  men  other | 55.7%  44.2%  .1% |
| Education | lower education  medium education  higher education | 12.5%  24.6%  62.7% |
| Migration background | western;  non-western;  no migration background | 2.1%  2.2%:  95.7%: |
| Living situation | Living alone | 22.3% |
| Work situation | Being employed with contract | 41.2% |
|  | Being self-employed | 9.5% |
| Having a health vulnerability | yes | 25.7% |

**Appendix 5**

***Overview of usage of the news information channels within different socio-demographic groups, in different data measurement rounds***

|  |  | **Gender** | | **Age categories** | | | | | | **Education level** | | | **Living situation** | | **Migration background** | | | **Health vulnerability** | | **Trust in government** | | |
| --- | --- | --- | --- | --- | --- | --- | --- | --- | --- | --- | --- | --- | --- | --- | --- | --- | --- | --- | --- | --- | --- | --- |
|  | Round | woman | man | 16-24 years | 25-39 years | 40-54 years | 55-69 years | 70-84 years | 85+ years | low | medium | high | alone | not alone | no | Western | not Western | no | yes | low trust | medium trust | high trust |
| General news consumption (frequency) | 1 | 3,22 | 3,23 | 2,88 | 3,11 | 3,24 | 3,33 | 3,35 | 3,32 | 3,22 | 3,21 | 3,24 | 3,29 | 3,16 | 3,18 | 3,21 | 3,29 | 3,19 | 3,25 | 3,16 | 3,17 | 3,34 |
|  | 2 | 3,15 | 3,15 | 2,88 | 2,98 | 3,10 | 3,25 | 3,30 | 3,35 | 3,13 | 3,15 | 3,17 | 3,20 | 3,09 | 3,11 | 3,15 | 3,18 | 3,11 | 3,19 | 3,07 | 3,09 | 3,28 |
|  | 3 | 2,76 | 2,83 | 2,37 | 2,52 | 2,75 | 2,92 | 3,01 | 3,02 | 2,79 | 2,77 | 2,83 | 2,80 | 2,73 | 2,75 | 2,82 | 2,82 | 2,75 | 2,84 | 2,59 | 2,74 | 2,97 |
|  | 4 | 2,47 | 2,60 | 2,04 | 2,24 | 2,51 | 2,77 | 2,90 | 2,85 | 2,49 | 2,51 | 2,59 | 2,58 | 2,53 | 2,50 | 2,57 | 2,54 | 2,49 | 2,58 | 2,42 | 2,46 | 2,78 |
|  | 5 | 2,32 | 2,45 | 2,35 | 2,20 | 2,64 | 2,68 | 2,71 | 2,59 | 2,33 | 2,37 | 2,46 | 2,50 | 2,55 | 2,31 | 2,42 | 2,43 | 2,34 | 2,43 | 2,33 | 2,49 | 2,77 |
|  | 6 | 2,96 | 2,93 | 2,64 | 2,81 | 2,99 | 3,20 | 3,26 | 2,99 | 2,91 | 2,92 | 3,00 | 3,04 | 2,92 | 2,93 | 2,91 | 3,00 | 2,90 | 2,99 | 2,87 | 2,96 | 3,11 |
|  | 7 | 3,17 | 3,11 | 2,90 | 2,95 | 3,07 | 3,22 | 3,29 | 3,53 | 3,13 | 3,11 | 3,18 | 3,21 | 3,10 | 3,11 | 3,13 | 3,19 | 3,11 | 3,16 | 3,08 | 3,12 | 3,28 |
|  | 8 | 2,97 | 2,97 | 2,64 | 2,75 | 2,94 | 3,11 | 3,22 | 3,27 | 2,97 | 2,95 | 2,99 | 3,05 | 2,92 | 2,93 | 3,01 | 2,97 | 2,93 | 3,01 | 2,73 | 3,01 | 3,22 |
|  | 9 | 2,92 | 2,93 | 2,58 | 2,68 | 2,88 | 3,07 | 3,16 | 3,21 | 2,89 | 2,89 | 2,98 | 2,99 | 2,86 | 2,91 | 2,92 | 2,94 | 2,90 | 2,95 | 2,81 | 2,90 | 3,07 |
|  | 10 | 2,81 | 2,87 | 2,52 | 2,60 | 2,78 | 3,00 | 3,10 | 3,13 | 2,82 | 2,80 | 2,90 | 2,90 | 2,80 | 2,81 | 2,87 | 2,84 | 2,81 | 2,87 | 2,67 | 2,86 | 3,01 |
|  | 11 | 2,84 | 2,90 | 2,45 | 2,65 | 2,81 | 3,09 | 3,19 | 3,24 | 2,84 | 2,83 | 2,93 | 2,96 | 2,84 | 2,79 | 2,93 | 2,88 | 2,85 | 2,88 | 2,70 | 2,92 | 3,09 |
|  | 12 | 2,68 | 2,77 | 2,40 | 2,49 | 2,70 | 2,95 | 3,00 | 3,04 | 2,71 | 2,67 | 2,80 | 2,82 | 2,70 | 2,65 | 2,79 | 2,74 | 2,71 | 2,74 | 2,59 | 2,76 | 2,95 |
|  | 13 | 2,52 | 2,65 | 2,51 | 2,42 | 2,58 | 2,75 | 2,75 | 2,78 | 2,52 | 2,54 | 2,69 | 2,68 | 2,59 | 2,48 | 2,63 | 2,64 | 2,56 | 2,61 | 2,36 | 2,63 | 2,91 |
|  | 14 | 2,49 | 2,57 | 2,23 | 2,31 | 2,56 | 2,79 | 2,79 | 2,74 | 2,45 | 2,46 | 2,67 | 2,62 | 2,53 | 2,44 | 2,68 | 2,46 | 2,52 | 2,54 | 2,40 | 2,54 | 2,78 |
|  | 15 | 2,28 | 2,39 | 1,99 | 2,05 | 2,33 | 2,55 | 2,63 | 2,59 | 2,26 | 2,28 | 2,46 | 2,40 | 2,31 | 2,28 | 2,39 | 2,33 | 2,29 | 2,37 | 2,15 | 2,34 | 2,58 |
|  | 16 | 2,27 | 2,37 | 1,87 | 2,07 | 2,35 | 2,58 | 2,63 | 2,56 | 2,24 | 2,27 | 2,45 | 2,38 | 2,31 | 2,27 | 2,32 | 2,37 | 2,29 | 2,35 | 2,21 | 2,30 | 2,52 |
|  | 19 | 2,04 | 2,20 | 1,89 | 1,85 | 2,06 | 2,29 | 2,41 | 2,41 | 2,09 | 2,08 | 2,19 | 2,18 | 2,12 | 2,06 | 2,18 | 2,12 | 2,07 | 2,17 | 1,99 | 2,08 | 2,39 |
|  | 21 | 1,25 | 1,44 | 0,78 | 0,93 | 1,20 | 1,51 | 1,71 | 1,73 | 1,30 | 1,28 | 1,46 | 1,32 | 1,30 | 1,42 | 1,28 | 1,34 | 1,27 | 1,42 | 1,20 | 1,20 | 1,53 |
|  | Average | 2,54 | 2,61 | 2,27 | 2,34 | 2,56 | 2,77 | 2,85 | 2,84 | 2,53 | 2,53 | 2,65 | 2,65 | 2,56 | 2,53 | 2,60 | 2,59 | 2,54 | 2,61 | 2,44 | 2,58 | 2,80 |
| TV news (% respondents) | 1 | 73% | 73% | 60% | 61% | 71% | 78% | 82% | 84% | 71% | 74% | 74% | 75% | 71% | 79% | 68% | 71% | 72% | 74% | 66% | 73% | 80% |
|  | 3 | 66% | 67% | 48% | 51% | 65% | 76% | 81% | 77% | 65% | 67% | 67% | 67% | 65% | 71% | 63% | 66% | 66% | 67% | 57% | 67% | 75% |
|  | 5 | 56% | 59% | 38% | 38% | 54% | 67% | 75% | 73% | 56% | 58% | 59% | 58% | 57% | 62% | 54% | 58% | 57% | 58% | 48% | 56% | 68% |
|  | 6 | 64% | 65% | 48% | 49% | 59% | 72% | 78% | 80% | 63% | 65% | 65% | 65% | 64% | 71% | 58% | 64% | 63% | 66% | 60% | 65% | 71% |
|  | 7 | 69% | 70% | 52% | 54% | 68% | 78% | 83% | 84% | 68% | 70% | 71% | 71% | 69% | 75% | 66% | 68% | 70% | 70% | 64% | 71% | 75% |
|  | 8 | 64% | 64% | 49% | 46% | 62% | 72% | 77% | 79% | 62% | 65% | 66% | 65% | 63% | 70% | 59% | 64% | 64% | 65% | 52% | 67% | 74% |
|  | 9 | 68% | 69% | 54% | 51% | 65% | 77% | 82% | 83% | 67% | 69% | 70% | 70% | 68% | 73% | 63% | 69% | 68% | 69% | 61% | 71% | 74% |
|  | 10 | 67% | 68% | 52% | 52% | 66% | 75% | 80% | 78% | 65% | 68% | 68% | 68% | 66% | 73% | 62% | 67% | 67% | 68% | 58% | 69% | 74% |
|  | 19 | 50% | 57% | 44% | 37% | 47% | 58% | 66% | 69% | 52% | 54% | 55% | 54% | 53% | 53% | 51% | 56% | 53% | 54% | 42% | 55% | 63% |
|  | Average | 64% | 66% | 49% | 49% | 62% | 73% | 78% | 79% | 63% | 65% | 66% | 66% | 64% | 70% | 60% | 65% | 64% | 66% | 56% | 66% | 73% |
| Newspapers and local media (% respondents) | 1 | 60% | 65% | 52% | 51% | 59% | 68% | 73% | 74% | 58% | 63% | 68% | 65% | 60% | 69% | 62% | 57% | 63% | 62% | 58% | 64% | 67% |
|  | 3 | 54% | 60% | 44% | 43% | 52% | 64% | 71% | 69% | 52% | 56% | 63% | 59% | 56% | 63% | 57% | 52% | 58% | 57% | 51% | 58% | 62% |
|  | 5 | 49% | 56% | 34% | 36% | 46% | 60% | 67% | 69% | 46% | 52% | 58% | 53% | 51% | 58% | 51% | 47% | 53% | 51% | 44% | 52% | 60% |
|  | 6 | 59% | 65% | 50% | 48% | 55% | 68% | 73% | 77% | 58% | 60% | 67% | 64% | 60% | 67% | 63% | 55% | 62% | 62% | 62% | 62% | 65% |
|  | 7 | 61% | 66% | 49% | 48% | 58% | 71% | 76% | 80% | 60% | 62% | 68% | 65% | 62% | 69% | 62% | 60% | 64% | 63% | 59% | 66% | 66% |
|  | 8 | 57% | 62% | 46% | 43% | 52% | 64% | 71% | 78% | 54% | 59% | 64% | 61% | 57% | 65% | 58% | 56% | 60% | 59% | 50% | 62% | 65% |
|  | 9 | 59% | 64% | 49% | 46% | 55% | 66% | 72% | 79% | 56% | 60% | 67% | 63% | 59% | 67% | 59% | 57% | 61% | 61% | 56% | 62% | 65% |
|  | 10 | 54% | 60% | 45% | 43% | 51% | 63% | 70% | 69% | 52% | 55% | 64% | 59% | 55% | 63% | 54% | 54% | 58% | 56% | 52% | 59% | 60% |
|  | 19 | 40% | 51% | 34% | 27% | 36% | 51% | 61% | 65% | 41% | 44% | 52% | 47% | 44% | 51% | 41% | 45% | 46% | 46% | 39% | 48% | 51% |
|  | Average | 55% | 61% | 45% | 43% | 52% | 64% | 70% | 73% | 53% | 57% | 64% | 60% | 56% | 64% | 56% | 54% | 58% | 57% | 52% | 59% | 62% |
| Governmental channels (% respondents) | 1 | 44% | 39% | 50% | 52% | 49% | 43% | 30% | 23% | 40% | 41% | 43% | 42% | 41% | 37% | 44% | 43% | 42% | 41% | 41% | 40% | 42% |
|  | 3 | 33% | 30% | 39% | 39% | 38% | 34% | 24% | 15% | 30% | 31% | 33% | 33% | 30% | 27% | 38% | 29% | 29% | 34% | 30% | 30% | 34% |
|  | 5 | 28% | 25% | 26% | 33% | 34% | 32% | 22% | 13% | 25% | 27% | 28% | 27% | 26% | 21% | 36% | 25% | 25% | 28% | 22% | 25% | 32% |
|  | 6 | 39% | 35% | 45% | 48% | 43% | 39% | 29% | 21% | 35% | 37% | 40% | 38% | 36% | 32% | 44% | 36% | 35% | 40% | 36% | 36% | 41% |
|  | 7 | 42% | 36% | 50% | 49% | 47% | 41% | 27% | 22% | 35% | 39% | 43% | 41% | 38% | 37% | 46% | 34% | 37% | 41% | 36% | 38% | 44% |
|  | 8 | 36% | 33% | 43% | 43% | 41% | 37% | 28% | 15% | 32% | 35% | 37% | 36% | 33% | 31% | 43% | 30% | 32% | 37% | 28% | 35% | 40% |
|  | 9 | 32% | 29% | 33% | 39% | 35% | 34% | 24% | 19% | 28% | 31% | 34% | 31% | 30% | 29% | 38% | 25% | 29% | 32% | 26% | 30% | 36% |
|  | 10 | 36% | 35% | 36% | 41% | 39% | 38% | 29% | 30% | 33% | 35% | 38% | 36% | 35% | 31% | 46% | 29% | 33% | 38% | 30% | 35% | 41% |
|  | 19 | 25% | 23% | 31% | 26% | 26% | 25% | 21% | 15% | 22% | 24% | 26% | 26% | 22% | 22% | 29% | 21% | 22% | 26% | 21% | 22% | 28% |
|  | Average | 35% | 32% | 39% | 41% | 39% | 36% | 26% | 19% | 31% | 33% | 36% | 34% | 32% | 30% | 40% | 30% | 32% | 35% | 30% | 32% | 38% |
| Medical channels (% respondents) |  | 7% | 6% | 7% | 7% | 6% | 6% | 7% | 5% | 5% | 7% | 7% | 7% | 6% | 6% | 6% | 7% | 5% | 8% | 9% | 5% | 5% |
|  | 3 | 4% | 4% | 4% | 4% | 4% | 3% | 3% | 3% | 3% | 4% | 4% | 4% | 4% | 3% | 4% | 4% | 3% | 5% | 4% | 4% | 4% |
|  | 5 | 4% | 4% | 6% | 4% | 4% | 4% | 4% | 2% | 4% | 4% | 4% | 4% | 4% | 3% | 3% | 6% | 3% | 5% | 4% | 4% | 4% |
|  | 6 | 4% | 4% | 6% | 4% | 4% | 4% | 4% | 2% | 4% | 4% | 4% | 4% | 4% | 3% | 3% | 6% | 3% | 5% | 5% | 4% | 4% |
|  | 7 | 4% | 4% | 4% | 4% | 3% | 4% | 4% | 6% | 4% | 4% | 5% | 4% | 4% | 4% | 4% | 5% | 3% | 5% | 4% | 5% | 4% |
|  | 8 | 3% | 4% | 4% | 4% | 4% | 4% | 4% | 2% | 3% | 4% | 4% | 4% | 3% | 3% | 4% | 4% | 3% | 5% | 4% | 3% | 4% |
|  | 9 | 4% | 4% | 5% | 3% | 3% | 3% | 4% | 5% | 4% | 4% | 4% | 4% | 4% | 4% | 3% | 4% | 3% | 5% | 4% | 3% | 4% |
|  | 10 | 5% | 6% | 4% | 5% | 5% | 6% | 7% | 6% | 5% | 5% | 5% | 5% | 5% | 4% | 6% | 6% | 4% | 7% | 6% | 5% | 5% |
|  | 19 | 3% | 3% | 3% | 3% | 3% | 2% | 3% | 5% | 3% | 3% | 3% | 3% | 3% | 2% | 3% | 4% | 3% | 4% | 4% | 3% | 3% |
|  | Average | 4% | 4% | 5% | 4% | 4% | 4% | 4% | 4% | 4% | 4% | 4% | 4% | 4% | 4% | 4% | 5% | 3% | 5% | 5% | 4% | 4% |
| Online news websites (% respondents) | 1 | 47% | 52% | 65% | 61% | 59% | 50% | 36% | 27% | 41% | 49% | 59% | 52% | 47% | 53% | 49% | 47% | 50% | 49% | 44% | 50% | 55% |
|  | 3 | 43% | 48% | 63% | 61% | 57% | 46% | 29% | 20% | 37% | 45% | 55% | 47% | 44% | 47% | 47% | 43% | 46% | 46% | 40% | 46% | 52% |
|  | 5 | 39% | 38% | 51% | 52% | 52% | 41% | 25% | 16% | 39% | 39% | 38% | 39% | 39% | 37% | 39% | 40% | 37% | 40% | 46% | 35% | 35% |
|  | 6 | 46% | 51% | 66% | 61% | 58% | 46% | 31% | 26% | 39% | 48% | 57% | 50% | 47% | 51% | 46% | 48% | 48% | 48% | 44% | 49% | 52% |
|  | 7 | 49% | 54% | 72% | 64% | 59% | 50% | 34% | 27% | 42% | 50% | 61% | 52% | 50% | 54% | 48% | 51% | 52% | 50% | 50% | 50% | 53% |
|  | 8 | 43% | 48% | 65% | 59% | 56% | 46% | 30% | 18% | 38% | 45% | 54% | 47% | 44% | 49% | 42% | 46% | 46% | 46% | 37% | 48% | 52% |
|  | 9 | 45% | 48% | 66% | 60% | 57% | 44% | 30% | 21% | 39% | 45% | 55% | 48% | 44% | 50% | 45% | 44% | 47% | 45% | 46% | 46% | 47% |
|  | 10 | 45% | 49% | 70% | 60% | 57% | 45% | 31% | 20% | 40% | 46% | 56% | 49% | 46% | 48% | 48% | 45% | 48% | 47% | 44% | 48% | 49% |
|  | 19 | 33% | 38% | 55% | 45% | 44% | 35% | 24% | 12% | 28% | 34% | 44% | 36% | 36% | 38% | 36% | 33% | 36% | 35% | 33% | 36% | 39% |
|  | Average | 43% | 47% | 64% | 58% | 55% | 45% | 30% | 21% | 38% | 45% | 53% | 47% | 44% | 47% | 44% | 44% | 46% | 45% | 43% | 45% | 48% |
| Radio (% respodents) | 1 | 28% | 28% | 24% | 23% | 24% | 27% | 32% | 27% | 25% | 27% | 26% | 26% | 26% | 27% | 29% | 22% | 27% | 25% | 22% | 27% | 29% |
|  | 3 | 21% | 26% | 24% | 22% | 22% | 24% | 26% | 23% | 22% | 24% | 25% | 22% | 25% | 24% | 26% | 21% | 25% | 22% | 20% | 23% | 28% |
|  | 5 | 17% | 21% | 14% | 17% | 18% | 21% | 24% | 21% | 16% | 20% | 21% | 18% | 20% | 21% | 20% | 17% | 20% | 18% | 15% | 19% | 23% |
|  | 6 | 21% | 24% | 19% | 19% | 20% | 22% | 25% | 27% | 20% | 22% | 24% | 22% | 23% | 24% | 22% | 20% | 23% | 21% | 18% | 23% | 26% |
|  | 7 | 21% | 25% | 18% | 21% | 24% | 27% | 28% | 23% | 20% | 24% | 26% | 23% | 24% | 26% | 24% | 20% | 25% | 22% | 18% | 25% | 26% |
|  | 8 | 23% | 27% | 26% | 21% | 23% | 24% | 27% | 30% | 23% | 26% | 26% | 25% | 25% | 26% | 27% | 22% | 26% | 24% | 20% | 26% | 29% |
|  | 9 | 22% | 25% | 26% | 20% | 20% | 22% | 24% | 27% | 20% | 24% | 26% | 23% | 24% | 26% | 24% | 19% | 24% | 22% | 20% | 24% | 26% |
|  | 10 | 19% | 23% | 20% | 20% | 20% | 22% | 25% | 23% | 19% | 21% | 24% | 21% | 22% | 23% | 22% | 18% | 22% | 20% | 18% | 23% | 24% |
|  | 19 | 11% | 16% | 13% | 11% | 13% | 15% | 18% | 12% | 12% | 13% | 15% | 12% | 15% | 17% | 14% | 10% | 14% | 13% | 9% | 14% | 17% |
|  | Average | 20% | 24% | 20% | 19% | 20% | 23% | 25% | 24% | 20% | 22% | 24% | 21% | 23% | 24% | 23% | 19% | 23% | 21% | 18% | 23% | 25% |
| (online) Interpersonal Communication (% of respondents) | 1 | 51% | 48% | 71% | 62% | 55% | 45% | 35% | 31% | 50% | 50% | 49% | 50% | 50% | 50% | 50% | 50% | 50% | 50% | 53% | 49% | 48% |
|  | 3 | 47% | 44% | 74% | 56% | 51% | 40% | 31% | 22% | 47% | 45% | 45% | 45% | 46% | 44% | 46% | 48% | 46% | 46% | 53% | 42% | 43% |
|  | 5 | 39% | 38% | 59% | 46% | 44% | 36% | 27% | 20% | 39% | 39% | 38% | 39% | 39% | 37% | 39% | 40% | 37% | 40% | 46% | 35% | 35% |
|  | 6 | 46% | 44% | 68% | 58% | 48% | 39% | 29% | 28% | 45% | 46% | 44% | 45% | 45% | 45% | 50% | 40% | 44% | 46% | 48% | 42% | 44% |
|  | 7 | 46% | 42% | 71% | 53% | 50% | 40% | 31% | 19% | 45% | 44% | 43% | 44% | 44% | 45% | 43% | 44% | 43% | 45% | 46% | 42% | 44% |
|  | 8 | 45% | 41% | 70% | 53% | 49% | 38% | 29% | 20% | 44% | 44% | 41% | 42% | 44% | 42% | 46% | 42% | 43% | 44% | 46% | 41% | 43% |
|  | 9 | 33% | 32% | 55% | 42% | 37% | 27% | 19% | 16% | 35% | 32% | 31% | 31% | 34% | 35% | 31% | 32% | 32% | 33% | 37% | 31% | 30% |
|  | 10 | 35% | 34% | 58% | 44% | 39% | 29% | 23% | 13% | 37% | 34% | 32% | 34% | 35% | 35% | 33% | 35% | 34% | 35% | 38% | 32% | 32% |
|  | 19 | 27% | 28% | 45% | 30% | 29% | 23% | 19% | 20% | 29% | 28% | 27% | 26% | 29% | 28% | 28% | 27% | 27% | 28% | 32% | 24% | 27% |
|  | Average | 37% | 36% | 55% | 43% | 40% | 32% | 26% | 20% | 37% | 36% | 35% | 36% | 37% | 36% | 37% | 35% | 36% | 36% | 39% | 34% | 35% |

Note. Highlighted cells represent the most used news information channels, on average, within a socio-demographic group

**Appendix 6**

*Robustness analyses: overview of the substantial odd ratios in the five preselected data collection rounds*

| TV news | | | | | | (online) Interpersonal | | | | | Newspapers | | | | | Government | | | | | | Medical | | | | | | Online news | | | | | Radio | | | | | |
| --- | --- | --- | --- | --- | --- | --- | --- | --- | --- | --- | --- | --- | --- | --- | --- | --- | --- | --- | --- | --- | --- | --- | --- | --- | --- | --- | --- | --- | --- | --- | --- | --- | --- | --- | --- | --- | --- | --- |
|  | 1 | 5 | 8 | 10 | 19 | 1 | 5 | 8 | 10 | 19 | 1 | 5 | 8 | 10 | 19 | 1 | 5 | 8 | 10 | 19 | 1 | | 5 | 8 | 10 | 19 | 1 | | 5 | 8 | 10 | 19 | 1 | 5 | 8 | 10 | 19 |  |
| Gender |  |  |  |  | .72 |  |  |  |  |  |  |  |  |  | .63 |  |  |  |  |  |  | |  |  |  |  |  | |  |  |  |  |  |  |  |  |  |  |
| Age | 1.66 | 1.76 | 1.67 | 1.60 | 1.43 | .70 | .73 | .69 | .70 |  | 1.42 | 1.61 | 1.47 | 1.46 | 1.73 |  |  |  |  |  |  | |  |  |  |  | .63 | | .66 | .62 | .62 | .68 |  |  |  |  |  |  |
| Education |  |  |  |  |  |  |  |  |  |  |  |  |  |  |  |  |  |  |  |  |  | |  |  |  |  | 1.43 | |  | 1.46 | 1.43 | 1.43 |  |  |  |  |  |  |
| Migr. not western | .58 |  | .70 | .69 |  |  |  |  |  |  | .60 | .67 | .73 | .66 |  |  |  | .54 | .50 | .67 |  | | 2.40 |  |  | 2.73 |  | |  |  |  |  |  |  |  |  | .65 |  |
| Migr,- western | .48 | .64 | .54 | .54 |  |  |  |  |  |  | .70 | .70 | .69 | .65 |  |  |  |  |  |  |  | |  |  |  |  |  | |  |  |  |  |  |  |  |  |  |  |
| Living alone | .72 |  |  |  |  |  |  |  |  |  |  |  |  |  |  |  |  |  |  |  |  | |  |  |  |  |  | |  |  |  |  |  |  |  |  |  |  |
| Employed |  |  |  |  |  |  |  |  |  |  |  |  |  |  |  |  |  |  |  |  |  | |  |  | .66 |  |  | |  |  |  |  |  |  |  |  |  |  |
| Self-employed |  |  |  |  |  |  |  |  |  |  |  |  |  |  |  |  |  |  |  |  |  | | . |  |  |  |  | |  |  |  |  |  |  |  |  |  |  |
| Health vulner. |  |  |  |  |  |  |  |  |  |  |  |  |  |  |  |  |  |  |  |  | 1.82 | | 1.87 | 1.96 | 1.99 | 1.52 |  | |  |  |  |  |  |  |  |  |  |  |
| Trust | 1.47 | 1.49 | 1.57 | 1.42 | 1.46 |  |  |  |  |  |  |  |  |  |  |  |  |  |  |  |  | |  |  |  |  |  | |  |  |  |  |  |  |  |  |  |  |

*Note.* Blank cells indicate unsubstantial odd ratios values, either >.70 or < .1.40.
